# Real-World Data on Detection of Germline and Somatic Pathogenic/Likely Pathogenic Variants in *BRCA1/2* and Other Susceptibility Genes in Ovarian Cancer Patients Using Next Generation Sequencing

**DOI:** 10.3390/cancers14061434

**Published:** 2022-03-10

**Authors:** Vida Stegel, Ana Blatnik, Erik Škof, Vita Šetrajčič Dragoš, Mateja Krajc, Brigita Gregorič, Petra Škerl, Ksenija Strojnik, Gašper Klančar, Marta Banjac, Janez Žgajnar, Maja Ravnik, Srdjan Novaković

**Affiliations:** 1 Department of Molecular Diagnostics, Institute of Oncology Ljubljana, Zaloška 2, 1000 Ljubljana, Slovenia; vstegel@onko-i.si (V.S.); vsetrajcic@onko-i.si (V.Š.D.); pskerl@onko-i.si (P.Š.); gklancar@onko-i.si (G.K.); 2 Cancer Genetics Clinic, Institute of Oncology Ljubljana, Zaloška 2, 1000 Ljubljana, Slovenia; ablatnik@onko-i.si (A.B.); mkrajc@onko-i.si (M.K.); kstrojnik@onko-i.si (K.S.); mbanjac@onko-i.si (M.B.); 3 Department of Solid Cancer Treatment, Division of Medical Oncology, Institute of Oncology Ljubljana, Zaloška 2, 1000 Ljubljana, Slovenia; eskof@onko-i.si (E.Š.); bgregoric@onko-i.si (B.G.); 4 Department of Surgical Oncology, Institute of Oncology Ljubljana, Zaloška 2, 1000 Ljubljana, Slovenia; jzgajnar@onko-i.si; 5 Department of Oncology, University Medical Centre Maribor, Ljubljanska ulica 5, 2000 Maribor, Slovenia; maja.ravnik@ukc-mb.si

**Keywords:** BRCA, HBOC, ovarian cancer, tumor genotyping, germline genotyping, PARP inhibitors, reverse mutation

## Abstract

**Simple Summary:**

Genotyping of BRCA genes is required for treatment with PARP inhibitors. Purposing to address treatment needs and familial cancer risk, the aim of our study was to introduce the most appropriate testing workflow in epithelial ovarian cancer patients (EOC) using germline and tumor genotyping of BRCA and other hereditary breast and/or ovarian cancer (HBOC) susceptibility genes. In consecutive patients with advanced non-mucinous EOC, who responded to platinum-based chemotherapy, germline and tumor genotyping were performed using Illumina’s next generation sequencing (NGS) panels. Sensitivity of tumor genotyping for detection of germline pathogenic/likely pathogenic variants (PV/LPV) was 96.2% for BRCA and 93.3% for HBOC genes. With germline genotyping-only strategy, 58.8% of HBOC PV/LPV and 68.4% of BRCA PV/LPV were detected. By tumor genotyping-only, 96.1% of HBOC PV/LPV and 97.4% of BRCA PV/LPV were detected. Tumor genotyping first followed by germline genotyping detects nearly all germline and somatic PV/LPV in the shortest time.

**Abstract:**

Detection of germline and somatic pathogenic/likely pathogenic variants (PV/LPV) in BRCA genes is at the moment a prerequisite for use of PARP inhibitors in different treatment settings of different tumors. The aim of our study was to determine the most appropriate testing workflow in epithelial ovarian cancer (EOC) patients using germline and tumor genotyping of BRCA and other hereditary breast and/or ovarian cancer (HBOC) susceptibility genes. Consecutive patients with advanced non-mucinous EOC, who responded to platinum-based chemotherapy, were included in the study. DNA extracted from blood and FFPE tumor tissue were genotyped using NGS panels TruSightCancer/Hereditary and TruSight Tumor 170. Among 170 EOC patients, 21.8% had BRCA germline or somatic PV/LPV, and additionally 6.4% had PV/LPV in other HBOC genes. Sensitivity of tumor genotyping for detection of germline PV/LPV was 96.2% for BRCA genes and 93.3% for HBOC genes. With germline genotyping-only strategy, 58.8% of HBOC PV/LPV and 68.4% of BRCA PV/LPV were detected. By tumor genotyping-only strategy, 96.1% of HBOC PV/LPV and 97.4% of BRCA PV/LPV were detected. Genotyping of tumor first, followed by germline genotyping seems to be a reasonable approach for detection of PV/LPV in breast and/or ovarian cancer susceptibility genes in non-mucinous EOC patients.

## 1. Introduction

Ovarian cancer (OC) is the sixth leading cause of cancer death among Slovene women, according to the data from the Cancer Registry of Republic of Slovenia. The incidence of OC in Slovenia is slowly declining, with 130 new cases in 2016 [1]. The most important risk factor for ovarian cancer is a family history of breast and ovarian cancer, often in association with inherited variants in *BRCA1* and *BRCA2* as well as in other breast and/or ovarian cancer susceptibility genes [2].

Different subtypes of OC have diverse origins, genetic alterations, and clinic-pathological features [3]. Among epithelial ovarian cancer (EOC) tumors, high-grade serous carcinomas (HGSC) are highly aggressive, resulting in poor overall prognosis. Genetically, 95% of HGSC tumors harbor *TP53* pathogenic/likely pathogenic variants (PV/LPV) and more than 50% display homologous recombination deficiency [4,5]. According to early reports from the Cancer Genome Atlas data (TCGA), 20% of HGSC harbor a PV/LPV in the *BRCA1* or *BRCA2* gene, of which 17% are germline and 3% are somatic PV/LPV [4]. Some subsequent reports, including the registration study for Olaparib, report up to 50% of BRCA positive tumors in the population of patients with serous EOC [6]. In aforementioned subsequent studies reported percentage of somatic PV/LPV in BRCA genes ranged from 20% to 46% of all BRCA PV/LPV detected in EOC patients [6,7]. Pathogenic/likely pathogenic variants in *BRCA1* and *BRCA2* genes were also reported in endometroid and clear cell ovarian carcinoma in 15% (4/26) and 10% (2/19), respectively [5,8].

Genetic counseling and testing for germline variants in *BRCA1* and *BRCA2* and in other hereditary breast and/or ovarian cancer (HBOC) susceptibility genes was established when the significance of hereditary cancer screening and preventive measures was recognized. Furthermore, testing for PV/LPV in BRCA genes has also become important for personalized treatment in patients with epithelial ovarian carcinoma.

According to the 2020 American Society of Clinical Oncology (ASCO) guidelines, testing for germline variants in BRCA and other susceptibility genes should be performed in all patients with EOC regardless of the clinical features or family history of cancer [9]. In addition, in the Recommendation 1.1. of the very same guidelines, it is stated that “Somatic tumor testing for *BRCA1* and *BRCA2* pathogenic or likely pathogenic variants should be performed in women who do not carry a germline pathogenic or likely pathogenic *BRCA1/2* variant” [9].

European Society for Medical Oncology and European Society of Gynecological Oncology (ESMO-ESGO) consensus conference recommendations on ovarian cancer published in 2019 recognize that poly-ADP ribose polymerase (PARP) inhibitors have greatest activity in patients with PV/LPV in BRCA genes, and thus testing for *BRCA1/2* PV/LPV is recommended for all non-mucinous ovarian cancer [10]. They also suggest to consider testing for PV/LPV in other homologous recombination (HR) genes (particularly *RAD51C*, *RAD51D*, *BRIP1,* and *PALB2*), since studies have shown that tumors harboring PV/LPV in those HR genes respond to PARP inhibitors [10,11]. ESMO-ESGO recommendations also emphasize the importance of the detection of germline and somatic PV/LPV in BRCA genes, but give no precise recommendations on the testing strategy. In Slovenia, PARP inhibitors are prescribed as the first line treatment for advanced ovarian cancer (AOC) patients if the tumor harbors germline or somatic BRCA PV/LPV. Therefore, BRCA status should be determined in AOC patients before treatment with PARP inhibitors. When planning the testing strategy, it is important to recognize that 54–80% of all *BRCA1/2* PV/LPV in EOC tumors are germline, while the remaining variants are somatic [6,12]. Since patients benefit from treatment regardless whether variants are germline or somatic, it appears reasonable to test tumor tissue first, followed by germline testing from normal tissue (preferable from blood) for the purposes of genetic counseling. Tumor testing itself would theoretically reveal somatic as well as germline PV/LPV. However, the genotyping of tumor samples is challenging, since tumor tissue is most commonly preserved by formalin fixation causing DNA fragmentation, which may affect downstream genomic analyses [13]. In addition, around 50% of EOC have deficient homologous recombination, leading to extensive gains and losses of genomic material, which could additionally impair the detection of PV/LPVs in tumor tissue [4]. The aim of the study was to verify that formalin-fixed paraffin-embedded (FFPE) tumor tissue sequencing could reliably detect germline variants using matched tumor–blood samples. The study compares detected PV/LPV from tumors with the PV/LPV from blood samples of the same patients. It includes an assessment of efficiency of tumor and germline genotyping techniques to detect clinically actionable PV/LPV in BRCA genes and other hereditary breast and/or ovarian cancer susceptibility genes, as well as the most effective patient management pathways especially considering patients’ responsiveness to genetic counseling referral. The final aim was to introduce the most appropriate testing workflow, which considers the medical implications and capacities of genetic counseling services as well as the needs of medical oncologists for personalized treatment decisions.

## 2. Materials and Methods

### 2.1. Study Design

In the study, 170 consecutive patients, with either newly diagnosed advanced or recurrent non-mucinous epithelial ovarian cancer (non-mucinous EOC), including fallopian tube cancer and primary peritoneal serous cancers, who responded to platinum-based chemotherapy, were included from January 2019 to April 2020. Borderline EOC were not included in the study. All patients in this study were treated and tested at the Institute of Oncology, Ljubljana, Slovenia. The study was approved by National Ethics Committee (permission no. 100/05/15 on 27 July 2015 and permission no. 0120-303/218/3 on 13 June 2018) and Institutional Ethics Committee of Institute of Oncology Ljubljana (IOL) (permission no. ERID-KESOPKR/97 on 17 September 2015 and permission no. ERID-EK/136 on 21 May 2018), and registered at ISRCTN registry on 24 November 2015 (ISRCTN42408038). Patients’ samples were obtained after appropriate informed consents.

Inclusion and exclusion criteria were the same as described previously by our group [14]. Inclusion criteria were as follows: WHO performance status 0–1, histologically non-mucinous EOC, tubal or primary peritoneal cancer, availability of tumor tissue samples, indication for treatment with surgery (primary debulking or interval debulking) and chemotherapy with platinum agents (adjuvant or neoadjuvant or treatment of metastatic disease), normal kidney function (creatinine < 100 µmol/L, endogenous creatinine clearance (ECC) > 75 mL/L), and signed informed consent. Exclusion criteria were as follows: mucinous EOC, unavailability of FFPE tissue samples, WHO performance status ≥2, and surgery and/or platinum-based chemotherapy not indicated.

**Scheme 1:**
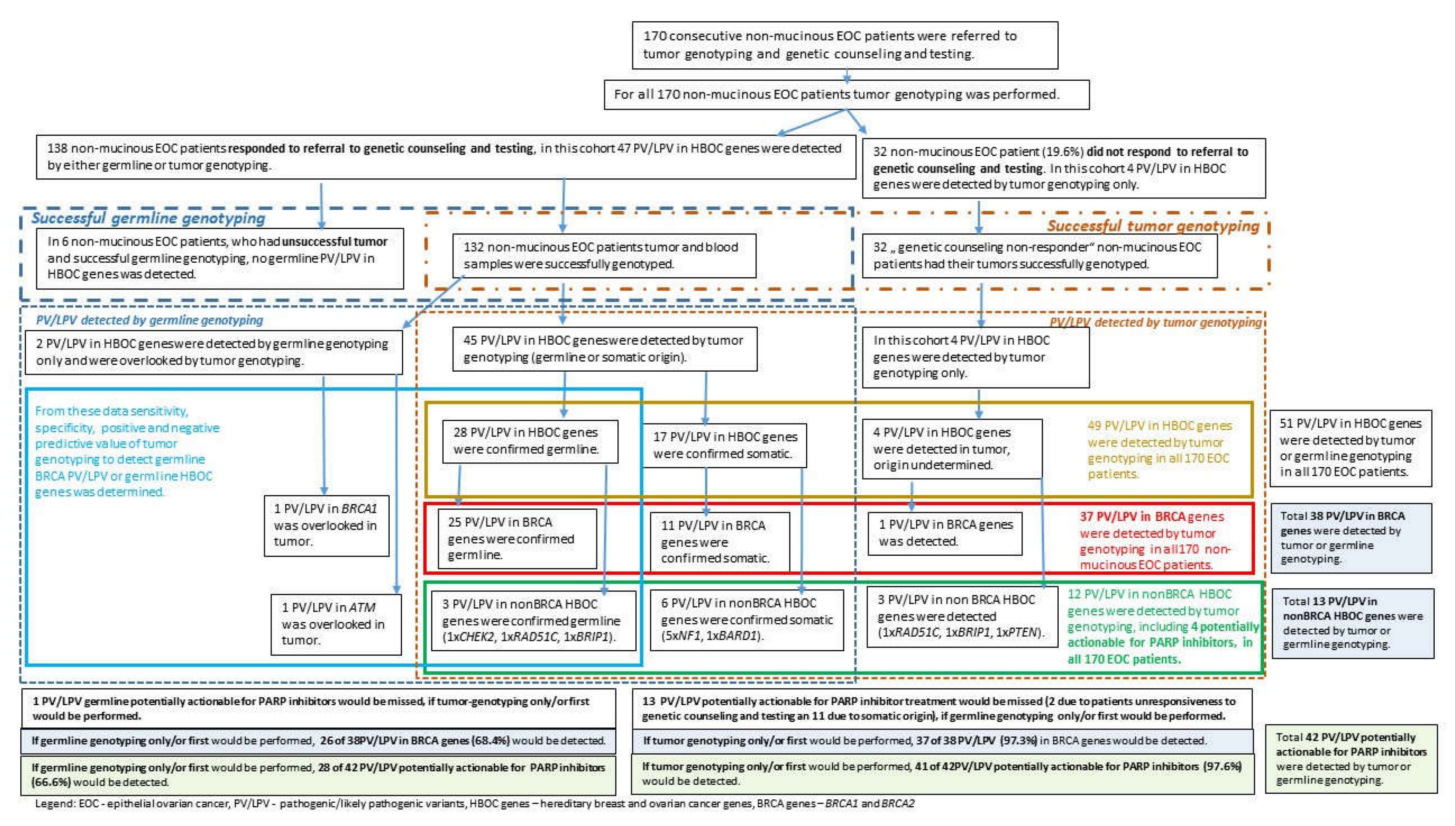
Study workflow.

All patients’ tumor samples were genotyped for the presence of *BRCA1* and *BRCA2* PV/LPV as well as for PV/LPV in other hereditary breast and/or ovarian cancer susceptibility genes. In parallel, all patients were referred to genetic counseling and germline testing for PV/LPV in hereditary breast and/or ovarian cancer susceptibility genes. Until the end of April 2020, 138 out of 170 patients with genotyped tumors underwent genetic counseling and germline genetic testing (group of matched tumor–germline samples) (Scheme 1). Family history of BRCA-related cancers (breast, ovarian, prostate, and pancreatic adenocarcinoma) was evaluated based on a questionnaire and reviewed by a clinical geneticist. Additionally, most diagnoses of cancer were verified in the Cancer Registry of Republic of Slovenia (http://www.slora.si/en, accessed on 15 May 2020), if possible. Genetic counseling was performed at the Department of Clinical Cancer Genetics IOL, while tumor and germline genetic testing was performed at the Department of Molecular Diagnostics IOL.

For this study, a virtual gene panel of 19 hereditary breast and/or ovarian cancer (HBOC) susceptibility genes was used for tumor and germline genotyping: *ATM* (LRG_135t1), *BARD1* (LRG_297t1), *BRCA1* (LRG_292t1), *BRCA2* (LRG_293t1), *BRIP1* (LRG_300t1), *CDH1* (LRG_301t1), *CHEK2* (LRG_302t1), *MLH1* (LRG_216t1), *MSH2* (LRG_218t1), *MSH6* (LRG_219t1), *NF1* (LRG_214t1), *NBN* (LRG_158t1), *PALB2* (LRG_308t1), *PMS2* (LRG_161t1), *PTEN* (LRG_311t1), *RAD51C* (LRG_314t1), *RAD51D* (LRG_516t1), *STK11* (LRG_319t1), and *TP53* (LRG_321t1).

Since data from the literature indicate that majority (95%) of HGSC tumors harbor *TP53* PV/LPV, the *TP53* gene was excluded from analysis. The narrowed-down targeted gene panel is referred to as “HBOC genes” and it includes all above-described breast and/or ovarian cancer susceptibility genes with the exception of the *TP53* gene. In the following content “BRCA genes” include only the *BRCA1* and *BRCA2* genes.

### 2.2. Samples

Tumor samples were all formalin fixed paraffin embedded (FFPE), and were evaluated by a pathologist according to the established laboratory protocol at the Department of Pathology at the IOL or at the Clinic of Gynecology and Obstetrics, Ljubljana University Medical Centre, Slovenia. Sample evaluation comprised of histological examination and conformation of the tumor type, determination of the percentage of tumor cells in the sample, and selection of the representative tumor block for DNA isolation. From the selected tumor block 10 μm thick slides were cut; the first and last slide were eosin–hematoxylin stained and used to visualize the presence of tumor cells, and to guide macro-dissection of tumor area from the unstained slides.

Whole blood samples were collected in EDTA tubes.

### 2.3. DNA Isolation

According to the established laboratory protocol genomic DNA was isolated from a whole blood sample using InnuPREP Master Blood Kit (Analytik Jena, Jena, Germany), and from FFPE-tissue sample using GeneRead DNA FFPE Kit (Qiagen GmbH, Hilden, Germany) or MagMax DNA/RNA kit (ThermoFisher Scientific, Woodward, St. Austin, TX, USA). Extracted DNA was quantified using Qubit dsDNA HS Assay kit and fluorimeter Qubit 3.0. (both ThermoFisher Scientific, Eugene, OR, USA).

### 2.4. Next Generation Sequencing

#### 2.4.1. Tumor Genotyping

Next generation sequencing (NGS) of tumor DNA samples was performed on NextSeq 550 Sequencing System, using TruSight Tumor 170-DNA (Illumina, San Diego, CA, USA) library preparation kit. Bioinformatical analysis was performed as described previously by our group [15]. This panel includes all previously listed HBOC susceptibility genes, where all protein coding exons are included, with the exception of exon 1 and promotor region of *BRCA1* and *BRCA2* genes, and *NF1* and *PMS2* genes, which are only partially covered. Samples were successfully sequenced, if >93.5% of targeted regions were covered >250×. The laboratory sensitivity of the method is: (i) >95% for detection of single nucleotide variants (SNV) with variant allele frequency (VAF) > 5%; (ii) >95% for detection of small (2–11 bp) insertion/deletion variants (indels) with VAF > 10%. The laboratory sensitivity and specificity of the method for detection of indels larger than 10 bp was not assessed. The copy number variations (CNVs) in genes of a virtual gene panel was determined using normalized coverage data from TruSight Tumor 170 Local App (Illumina). For BRCA genes only, an intragenic exonic deletion was considered, if two adjacent exons of BRCA genes had fold change under 0.6.

Variants detected in tumor sample were classified for their clinical importance according to ACMG/AMP/ASCO guidelines [16]. Variants are described according to HGVS v20.05 nomenclature [17].

#### 2.4.2. Genotyping for Germline Alterations/Variants

Next generation sequencing of blood DNA samples was performed on Illumina MiSeqDx Sequencing System using TruSight Cancer Panel or TruSight Hereditary Panel (Illumina, San Diego, CA, USA) to enrich and sequence of all translated exons and ±25 bp flanking intronic regions of all HBOC genes, bioinformatics and copy number analysis were performed as described by our group previously [14,15]. Samples were successfully sequenced if >95% of targeted regions were covered >40×. NGS sequencing has 100% sensitivity and specificity for detection of heterozygous germline SNV and small indels (<10 bp) according to Sanger sequencing.

Germline variants were classified for their clinical importance according to ACMG/AMP guidelines [18,19]. Variants are described according to HGVS v20.05 nomenclature [17].

All PV/LPV germline variants in HBOC genes detected by NGS were additionally confirmed by Multiplex Ligation-dependent Probe Amplification analysis or Sanger sequencing.

### 2.5. Multiplex Ligation-Dependent Probe Amplification (MLPA) and Sanger Sequencing

MLPA was performed to confirm the presence of copy number variations (CNVs) in blood DNA samples—using SALSA P045-BRCA2/CHEK2 Probemix and/or SALSA P002-BRCA1 (both MRC-Holland, Amsterdam, The Netherlands). Direct Sanger sequencing was performed to confirm the presence of SNV and small indels in blood DNA samples. Sanger sequencing and MLPA were performed as described by our group previously [15].

### 2.6. Statistical Analysis

To validate whether tumor genotyping strategy can successfully detect germline PV/LPV in HBOC genes, matched tumor–blood samples with sufficient genotyping quality were included in the analysis. Blood genotyping was taken as the reference method for germline PV/LPV detection. Since germline as well as tumor genotyping give binary results (positive or negative for PV/LPV), the sensitivity, specificity, positive, and negative predictive values for tumor genotyping to detect germline PV/LPV or patients with germline PV/LPV were calculated.

Sensitivity, specificity and predictive values of positive and negative tumor genotyping result were calculated as follows. Sensitivity of tumor genotyping for detection of patient with germline PV/LPV = a/(a + c). Specificity of tumor genotyping for detection of patient with germline PV/LPV = d/(b + d). Predictive value of a positive tumor genotyping result to predict positive germline genotyping result = a/(a + b). Predictive value of a negative tumor genotyping result to predict negative germline genotyping result = d/(c + d). The meaning of symbols are; a—true positives for germline variants (cases that were positive by germline and tumor genotyping); b—false positives for germline variants (cases that were negative by germline genotyping and positive by tumor genotyping); c—false negatives for germline variants (cases that were positive by germline genotyping and negative by tumor genotyping); d—true negatives for germline variants (cases that were negative by germline and tumor genotyping).

We also performed an assessment of workloads associated with three different approaches to genetic testing in ovarian cancer patients, using descriptive statistics.

## 3. Results

In the current study, a total of 170 FFPE tumor specimens from consecutive patients with advanced non-mucinous EOC were genotyped for the presence of PV/LPV in BRCA genes and other pre-specified genes. At the time of data analysis, 81.2% (138/170) patients opted for genetic counseling and germline testing; 32 patients whose tumors were successfully genotyped did not undergo genetic counseling and germline testing. Since in six tumor samples, NGS sequencing did not meet the quality criteria threshold, the final number of patients with successfully tested matched tumor-blood samples was 132. Germline NGS testing was performed in 127 (out of 132 blood samples), while 5 blood samples were genotyped using Sanger sequencing only for BRCA PV/LPV detected in tumor (Scheme 1). In our pair-matched cohort 75% (99/132) had confirmed somatic PV/LPV in the *TP53* gene.

### 3.1. Frequencies of Germline and Somatic PV/LPV

Our entire study group was divided in three subgroups—1. subgroup of patients with unsuccessful tumor genotyping; 2. subgroup of patients with successfully matched tumor and germline genotyping; and 3. subgroup of non-responders to referral to genetic counseling and testing. In Table 1, Table 2 and Table 3, cumulative genotyping results are presented. Among all 170 patients included in the study, 51 PV/LPV in HBOC genes were found in 27.1% of patients either by tumor or germline genotyping (Table 1). Germline PV/LPV in HBOC genes were found in 17.1% of patients, while somatic PV/LPV were found in 8.8%. In 2.3% of patients, the origin of PV/LPV was unknown, since these patients did not attend genetic counseling and confirmatory germline testing (subgroup of non-responders). Two germline PV/LPV, found in two different patients (1.2% of 170), were missed by tumor genotyping (one patient with *BRCA1* large deletion and another patient with SNV in *ATM*) (Scheme 1, Table 1).

Five patients had more than one PV/LPV in HBOC genes detected in tumor samples. One patient had a germline *CHEK2* PV/LPV and a somatic PV/LPV in the *BRCA2* gene, one patient had one germline and one somatic PV/LPV in *BRCA2*, one patient had one germline PV/LPV in *BRCA1* and one somatic PV/LPV in the *NF1* gene (Table S1), one had two germline PV/LPV detected, in the *ATM* and *RAD51C* genes, and one patient had two somatic PV/LPV in the *NF1* gene (Table S2).

Among aforementioned PV/LPV detected in 170 tested patients in the study, PV/LPV in BRCA genes were found in 21.8% of patients (Table 2). Germline PV/LPV in BRCA genes were found in 15.3% of patients, while somatic PV/LPV were found in 6.5% of patients. In one patient somatic as well as germline PV/LPV in the *BRCA2* gene were found, while in one patient the origin of PV/LPV in the *BRCA1* gene was unknown (Table 2, Scheme 1, and Table S1).

In nonBRCA, HBOC genes PV/LPV were found in 6.4% of patients. (Table 3). Germline PV/LPV in nonBRCA HBOC genes were found in 1.7% of patients (in *RAD51C, ATM, BRIP1,* and *CHEK2*) and somatic PV/LPV in 2.9% of patients (five variants in *NF1*, and one variant in *BARD1*). In 1.7% of patients, the origin of PV/LPV in nonBRCA HBOC genes (in *RAD51C, BRIP1,* and *PTEN*) was unknown since these patients did not attend genetic counseling and confirmatory germline testing (subgroup of non-responders) (Scheme 1 and Table S2).

Retrospective analysis of the subgroup of “non-responders” revealed that 6 of them died less than 4 months after referral to genetic counselling and 13 of them visited genetic counselor after our study’s observation period of 4 months.

### 3.2. Proportion of Detected PV/LPV in All 170 Consecutive Advanced Non-Mucinous EOC Patients Depending on the Approach to Testing: Tumor Genotyping Only vs. Germline Genotyping Only

In total, 51 PV/LPV in HBOC genes were detected by tumor or germline genotyping in 170 patients. By tumor genotyping only, 49 of 51 (96.1%) PV/LPV would have been detected in 44 patients. Due to patients’ unresponsiveness to genetic counseling/testing (32 patients) and somatic origin of 17 PV/LPV, with germline genotyping only 30 of 51 (58.8%) PV/LPV in HBOC genes would have been detected in 29 patients (Scheme 1, Tables S1 and S2).

In BRCA genes, a total of 38 PV/LPV were detected by tumor or germline genotyping in a cohort of 170 patients. By tumor genotyping only, 37 of 38 (97.4%) PV/LPV would have been detected in 37 patients. With germline genotyping only, 26 of 38 (68.4%) PV/LPV would have been detected in 26 patients (Tables S1 and S2).

If PV/LPV in *RAD51C* and *BRIP1* genes, besides BRCA genes, are also considered as potentially actionable for treatment with PARP inhibitors, then a total of 42 potentially actionable PV/LPV were detected by tumor or germline genotyping. By tumor genotyping only, 41 of 42 (97.6%) potentially actionable PV/LPV would have been detected in 41 patients. With the germline genotyping only, 28 of 42 (66.6%) potentially actionable PV/LPV would have been detected in 28 patients (Scheme 1, Tables S1 and S2).

### 3.3. Frequencies of Germline and Somatic PV/LPV in HBOC Genes among Subgroup of 132 Advanced Non-Mucinous EOC Patients with Matched Successful Tumor and Germline Genotyping

In the subgroup of 132 patients with matched successful tumor and germline genotyping, 47 PV/LPV in HBOC genes were found in 31.8% of patients either by tumor or germline genotyping (Table 1). Germline PV/LPV in HBOC genes were found in 21.9% of patients, while somatic PV/LPV were found in 11.3% (Table 1). Two PV/LPV were found only by germline genotyping (Scheme 1, Tables S1 and S2). As mentioned previously, in three patients, both somatic and germline PV/LPV in HBOC genes were detected. Two germline PV/LPV were found in two patients, but were missed by tumor genotyping (one patient with *BRCA1* large deletion and another patient with SNV in *ATM*) (Scheme 1, Table 1, Tables S1 and S2). Among the aforementioned PV/LPV detected in 132 non-mucinous EOC patients with matched tumor–blood samples, PV/LPV in BRCA genes were found in 27.2% of patients (Table 2). Germline PV/LPV in BRCA genes were found in 19.5% of patients, while somatic PV/LPV were found in 8.3% of patients. As mentioned before, one of those patients had somatic and germline PV/LPV in *BRCA2* and the other somatic PV/LPV in *BRCA2* and germline PV/LPV in *CHEK2*. Ten patients had only somatic PV/LPV in BRCA genes. *BRCA1* germline PV/LPV were present in 77.0% of germline BRCA positive patients (20/26), while *BRCA2* germline PV/LPV were present in 23.0% of germline BRCA positive patients (6/26) (Table S1). Conversely, somatic PV/LPV variants in the *BRCA2* gene were present in 54.5% of somatic BRCA positive patients (6/11) while PV/LPV in the *BRCA1* gene were present in 45.4% (5/11) (Table S1, Scheme 1).

In the group of 132 EOC patients, 10 PV/LPV in nonBRCA HBOC genes were found in 6.0% of patients (Table 3). As mentioned before, four germline PV/LPV in nonBRCA HBOC genes were found in three patients (2.2%) (in *RAD51C*, *ATM*, *BRIP1*, and *CHEK2*). Six somatic PV/LPV in nonBRCA HBOC genes were found in five patients (3.8%) (five in *NF1*, and one in *BARD1*). One somatic PV/LPV in the *NF1* gene was found in patient harboring at the same time germline PV/LPV in *BRCA1*. In one patient two somatic PV/LPV in *NF1* were detected (Scheme 1, Tables S1 and S2).

### 3.4. Efficiency of Tumor Genotyping for Detection of Germline Variants

The efficiency of tumor genotyping to detect germline variants from tumor tissue regarding the type of variant was assessed in the group of 132 patients with successful paired tumor and germline genotyping.

Germline genotyping revealed 22 small PV/LPV variants (SNV and small 1–7 bp indels) and 4 large intragenic PV/LPV deletions in BRCA genes (Table S1). Additionally, four PV/LPV variants in nonBRCA genes were detected (Tables S1–S3).

Comparing tumor to germline genotyping, all germline small variants in BRCA genes were also detected in tumors, while one small variant in the *ATM* gene was missed (*ATM*: c.5932G > T p.(Glu1978*). Among germline large intragenic deletions in BRCA genes, three out of four variants were detected by tumor genotyping (Table S3). Altogether, 2 PV/LPV out of 30 germline PV/LPV in HBOC genes were not detected, using tumor testing (6.7%).

Sensitivity of the method used for genotyping of ovarian tumor FFPE samples was 100% for small germline PV/LPV in BRCA genes (all of 22 small variants were detected), but when taking into account all HBOC genes the sensitivity for small variant detection was 96.2% (25 cases of 26). The sensitivity of NGS method used for tumor genotyping for detection of germline large intragenic deletions in BRCA genes was 75.0% (three of four large deletions were detected). No large intragenic deletion was found in other nonBRCA HBOC genes. Sensitivity of FFPE ovarian tumor genotyping for detection of all kinds of germline PV/LPV (including SNV, small indels, and large intragenic deletions) was 96.2% (25/26) for BRCA genes and 93.3% (28/30) for all HBOC genes (Tables S1–S3). Identical sensitivity of FFPE ovarian tumor genotyping for detection/recognition of patients with germline PV/LPV in BRCA genes was determined: 96.2% (25/26) (Table 4, Table S1). However, in one patient with two germline PV/LPV in HBOC genes (in *RAD51C* and *ATM*) only one PV/LPV (in *RAD51C*) was detected. For the purposes of our analysis, we considered this patient as not having been recognized as a carrier of germline PV/LPV based on tumor genotyping. Consequently, the sensitivity of FFPE ovarian tumor genotyping for detection/recognition of patients with germline PV/LPV in all HBOC genes was determined to be 93.1% (27/29) (Table 5, Table S2).

Specificity of tumor genotyping for detection of patients with germline PV/LPV in BRCA genes is 90.6% (96/106) (Table 4), and for detection of patients with germline PV/LPV in HBOC genes 87.3% (90/103) (Table 5).

Positive predictive value of tumor genotyping for detection of patients with germline PV/LPV in BRCA genes was 71.4% (25/35) and for detection of patients with germline PV/LPV in HBOC genes 67.5% (27/40) (Table 4 and Table 5). Meaning that there is a 71.4% probability that PV/LPV in BRCA genes detected in the tumor are of germline origin, and a 67.5% probability that PV/LPV in HBOC genes detected in the tumor are of germline origin.

Negative predictive value of tumor genotyping for detection of patients with germline PV/LPV in BRCA genes was 98.9% (96/97) and for detection of patients with germline PV/LPV in HBOC genes 97.8% (90/92) (Table 4 and Table 5). Meaning, that there is a 98.9% probability that a patient who is negative for PV/LPV in BRCA genes by tumor genotyping will be negative also by germline genotyping, and a 97.8% probability that patients negative for PV/LPV in HBOC genes by tumor genotyping will be negative also by germline genotyping.

### 3.5. Family History of BRCA-Related Cancers among Matched Advanced Non-Mucinous EOC Patients

Family history of BRCA-related cancers was reported by the referring physician in 45 of 132 patients (34.1%). Thirty germline PV/LPV in at least one of the HBOC genes were detected in 29 of 132 patients (22.0%). Family history of HBOC was seen in 14 of 29 patients with germline PV/LPV (48.3%) (Tables S1 and S2).

Among 103 patients without germline or somatic PV/LPV in HBOC genes, a positive family history was reported in 27 cases (26.0%).

Somatic PV/LPV in BRCA genes were detected in tumors of 11 patients. One of them was also a carrier of germline PV/LPV in *BRCA2* and the other was also a carrier of germline PV/LPV in *CHEK2*. Therefore, there were 10 patients with only somatic BRCA PV/LPV, which represents 7.5% (10/132) of all matched advanced non-mucinous EOC patients. From this cohort of 10 patients, family history of HBOC was reported in 3 patients (33.3%). In other words, in a cohort of 132 matched advanced non-mucinous EOC patients 3 patients (2.3%) with only somatic BRCA PV/LPV also had a family history of HBOC, with one of them carrying a germline PV in the *CHEK2* gene.

Two advanced non-mucinous EOC patients, who carried germline PV/LPV (deletion of exons 1 and 2 in *BRCA1* and c.5932G > T in *ATM*), which were not detected in tumor testing, had a diagnosis of high grade serous ovarian cancer and no family history of breast and ovarian cancer. One patient, where a *BRCA1* PV/LPV was missed on tumor testing, had a family history of prostate cancer in a second-degree relative. The other patient, who had a germline PV/LPV in *RAD51C* (detected also in the tumor) and in the *ATM* gene (not detected in tumor), had no family history of cancer.

### 3.6. Analysis of Three Different Approaches to Germline and Tumor Testing

**Scheme 2:**
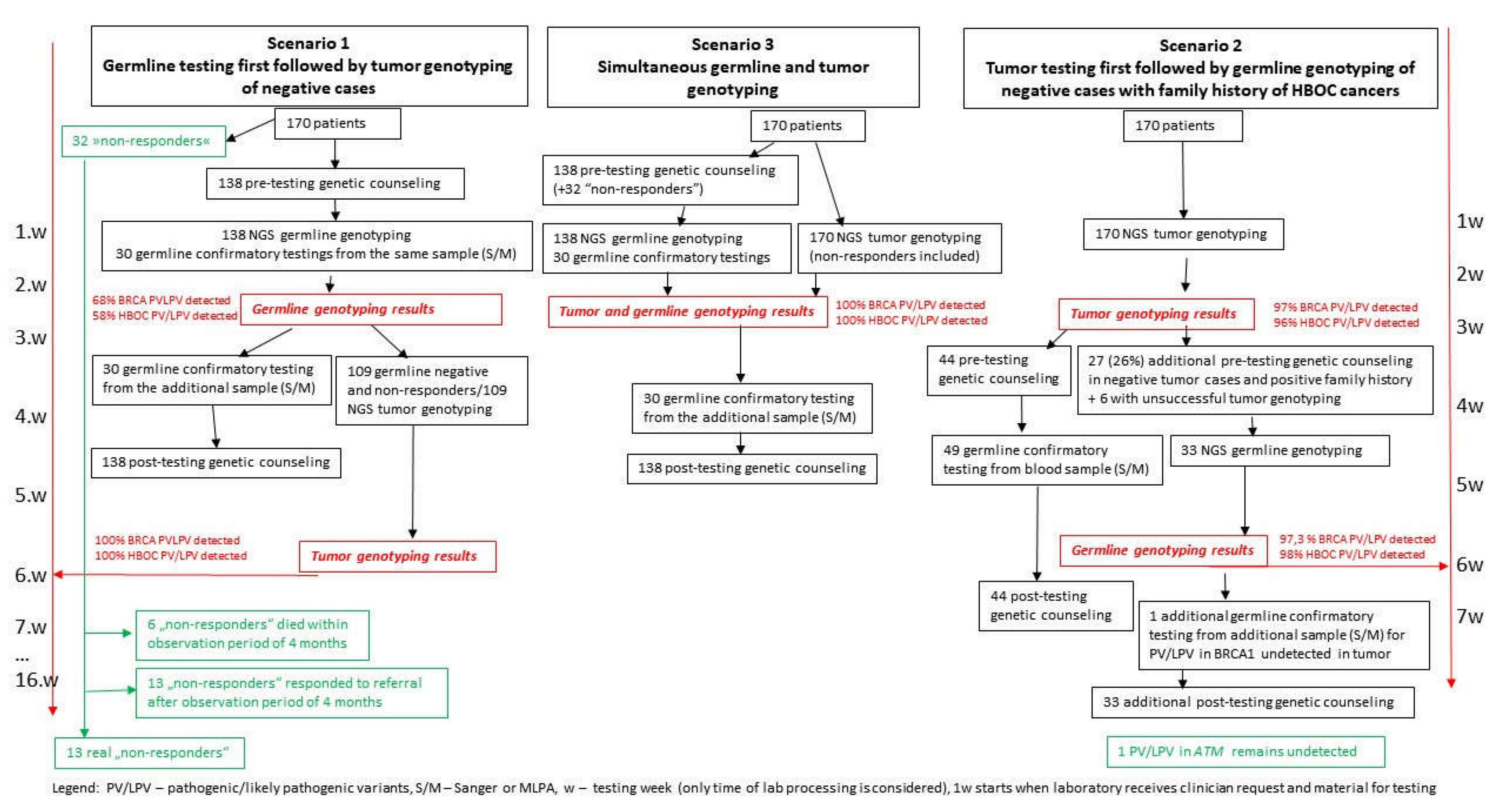
Comparison of testing scenarios—germline genotyping first vs. tumor genotyping first.

Additionally, we investigated three different approaches to genetic testing of ovarian cancer patients previously reported in the literature and assessed the workloads involved for the diagnostic laboratory and clinical genetics service. Data collected in our study were used to illustrate the detection rates and workloads in two possible hypothetical approaches, Scenario 1 and Scenario 2. Scenario 1 would involve testing all patients who opt for NGS germline testing from blood first. Those who received negative results and those who chose not to undergo germline testing within a 2–3-week timeframe would then be tested using NGS from tumor tissue (Scheme 2). In Scenario 2, all patients would be tested using NGS from tumor tissue first, and those with a positive result would have confirmatory non-NGS testing from blood. Those with negative tumor tissue results and a positive family or personal history indicative of HBOC would receive NGS germline testing. These two approaches were compared to Scenario 3, which involves simultaneous NGS testing from blood and tumor tissue, as performed in this study (Scheme 2). The results are presented in Table S4. Scenario 3 would result in all germline and somatic variants being detected within 2–3 weeks, but would entail 138 NGS germline tests and 170 NGS tumor tissue tests, with 138 pre-test genetic counselling appointments and additional posttest counseling for all positive responders. It would therefore be labor intensive and costly. Scenario 1 would reduce the number of tumor tissue NGS tests to 109, but due to the sequential nature of this approach (blood first, then tumor, if needed), only 68% of all PV/LPV would be detected within the 2–3-week timeframe. Scenario 2 would reduce the number of NGS tests from blood to 33, and the number of pre-test appointments to 77, thus reducing the workload and cost of testing, 97% of all PV/LPV would be detected within 2–3 weeks. The comparison of Scenario 1 and Scenario 2 is presented in Table S5.

## 4. Discussion

According to National Comprehensive Cancer Network (NCCN) Guidelines as well as ESMO-ESGO recommendations, all EOC patients, including patients with fallopian tube cancer and primary peritoneal cancer, should undergo germline and somatic tumor testing. Germline testing is required to identify patients with hereditary cancer predisposition and cascade testing. Since the introduction of PARP inhibitors, the *BRCA1/2* status (germline or somatic) became important also for personalized treatment decisions in these patients [20]. In addition to *BRCA1/2* testing, NCCN and ESMO-ESGO recommend considering somatic tumor testing for PV/LPV in other homologous deficiency (HR) genes as well as the evaluation of HR deficiency when deciding on PARP inhibitor therapy.

As mentioned in the Introduction, there are some remarkable clinical studies researching different angles related to treatment with PARP inhibitors in EOC patients [21]. There are also recommendations (NCCN, ESMO-ESGO) on which genes and what other parameters (genomic scars) should be determined to identify patients who will have significant benefit from PARP inhibitor therapy [10,22]. Unfortunately, neither of these studies, nor the NCCN and ESMO-ESGO guidelines, provide precise guidance on how to approach NGS testing to achieve optimal results. Consequently, different European countries have different NGS strategies for BRCA testing in ovarian cancer. According to accessible literature, in Spain, testing for BRCA germline PV/LPV is offered to all patients with high-grade non-mucinous ovarian carcinomas, followed by somatic testing if negative for germline PV/LPV [5]. Other countries only list which patients are tested, without giving information on whether they are testing first for germinal or somatic alterations [13,23].

To improve the workflow of NGS germline and somatic testing processes in terms of speed, efficiency, and optimal sampling, several questions should be addressed: should tumor and germline genotyping be performed in parallel or one after the other; what would be the optimal testing strategy for identifying the highest proportion of germline and somatic PV/LPV; and, finally, how to perform the required testing within optimal turnaround time frame? Thus, the objective of this article is to provide real world data on germline and tumor testing in advanced non-mucinous EOC patients, collected from January 2019 to April 2020. The final aim is to implement the most appropriate testing workflow covering the needs of genetic counseling as well as the needs of clinicians for treatment decisions.

Given the fact that germline testing for BRCA PV/LPV in individuals from HBOC syndrome families has been in use for a long time for genetic counseling purposes, it would be reasonable to offer germline testing as the first step. Since germline predisposition testing would only detect germline variants, it should be complemented with tumor testing of BRCA negative cases to detect somatic variants. The whole procedure of genetic counseling and germline predisposition testing followed by tumor testing is time consuming, and is based on the presumption that all non-mucinous EOC patients will show up for genetic counseling and testing. As showed in our study as well as in studies of other researchers, a certain proportion of patients do not respond to this referral during the observation period of 4 months [24].

On the other hand, NGS tumor testing would theoretically reveal somatic as well as germline BRCA PV/LPV, but the laboratory should be aware that non-mucinous EOC patients are also candidates for germline testing of PV/LPV in several genes other than BRCA. Based on the results of our study, we propose the optimal strategy is testing the tumor first using an NGS panel that includes all HBOC genes. NGS tumor testing should be able to detect all types of PV/LPV in HBOC genes, from SNV, and small indels to large intragenic deletions or duplications. In positive cases, tumor testing should be followed by conformational germline testing to clarify whether the variant is of germline or somatic origin.

In our study, 96.5% of tumor samples were successfully sequenced, which correlates very well with previous reports in Dutch EOC patient’s cohort (96.8%) and in Italian EOC patient’s cohort (99.0%) [7,25].

In our cohort of matched advanced non-mucinous EOC patients, 36 out of 132 patients (27.2%) were found by germline and tumor testing to have PV/LPV in BRCA genes. Most of the BRCA positive patients carried a germline BRCA PV/LPV (26; 69.4%) while 10 patients (29.7%) had only somatic BRCA PV/LPV (Table 2, Table S1, Scheme 1). Regardless of variant’s origin, PV/LPV in *BRCA1* (60.0%) were more prevalent than PV/LPV in *BRCA2* (40.0%). Our results agree with the reports of other authors who reported BRCA PV/LPV in 16.7–28.1% of non-mucinous EOC patients [6,7,8,12,25,26,27,28]. The percentage of BRCA positive patients in our study is closest to the results of Italian research group who reported BRCA PV/LPV in 28.1% of 221 non-mucinous non-borderline EOC cases. Among BRCA positive cases there were 78.3% of germline and 21.7% of somatic origin [25]. The lowest ratio of BRCA PV/LPV was reported by the Dutch research group who detected BRCA PV/LPV in 16.7% (germline in 56.8%, and somatic in 43.2%) of non-borderline EOC tumors [7].

The sensitivity of the used NGS method for detection of germline BRCA PV/LPV in FFPE tumor tissue varied between 100% (for small variants) and 75% (for large intragenic deletions). Actually, three of four large intragenic variants were detected by tumor genotyping and the only variant that was not identified, was the deletion of promotor region and exons 1 and 2 of the *BRCA1* gene (Table S1). The promoter region and exon 1 are not included in TruSight Tumor 170 library preparation kit; therefore, variants in these two regions cannot be detected. The detection of large intragenic exon copy number variants represents one of the major limitations of FFPE tumor genotyping of non-mucinous EOC tumors due to extensive tumor copy number aberrations caused by homologous recombination deficiency [29]. The other variant that was missed was not in *BRCA1/2* genes but in the *ATM* gene. In the tumor bearing the pathogenic variant *ATM*: c.5932G > T p. (Glu1978*), loss of heterozygosity (LOH) (enriching wild type allele) occurred and as a consequence, the variant frequency was below the limit of detection used in tumor genotyping (Table S2). However, it is worth mentioning the high negative predictive values of tumor genotyping for detection of germline PV/LPV in BRCA genes and other nonTP53-HBOC genes. Testing the tumor first could therefore reduce the number of germline tests for PV/LPV in these genes.

It is interesting that in the majority of patients carrying the germline BRCA PV/LPV (22 of 26 cases), the variant frequency of the PV/LPV was above 60.0% indicating that there was partial LOH (Supplementary Table S1). In one tumor sample (no. 13), two PV/LPV (one germline and one somatic) in the *BRCA2* gene were detected. Somatic PV/LPV was probably the second hit causing deactivation of the wild-type allele. Another patient from our cohort (sample no. 4) acquired a reverse PV in the *BRCA1* gene where the nucleotide change occurred at the same codon (at the same allele) as germline nonsense variant c.1687C > T in the *BRCA1* gene restoring the reading frame and causing an amino acid change (Table S1).

Based on the results of our study, with germline genotyping only, 15.3% of all patients included in the study (26/170) would qualify for PARP inhibitor therapy (patients with BRCA PV/LPV) in first line setting of ovarian cancer treatment (Table 2). With tumor genotyping, 21.8% of all patients included in the study (37/170) had BRCA PV/LPV (Table 2). Therefore, the number of eligible patients for therapy with PARP inhibitors in first line ovarian cancer treatment setting was increased by substantial 42.0% (11/26) when tumor genotyping was applied. British Gynecological Cancer Society and British association of Gynecological Pathologists came to a similar conclusion and reported that tumor genotyping identified 50% more patients with BRCA mutations than germline genotyping only, while germline genotyping identified 10% more BRCA mutations (large duplications and deletions), which were undetectable by tumor genotyping. Based on this, their recommendation was that germline and tumor genotyping should be performed in parallel [30]. Our analysis of possible approaches to testing indicates that offering tumor tissue testing first, followed by germline testing for PV/LPV detected in the tumor or additional NGS germline testing in cases of positive personal or family history for HBOC, would enable us to detect nearly all PV/LPV as quickly as with parallel blood/tumor testing whilst greatly reducing the cost and workload involved.

The real-world data from Slovene advanced non-mucinous EOC patients show that all of them had tumor genotyping and were simultaneously referred for genetic counselling and germline testing. However, 32 of 170 (18.8%) advanced non-mucinous EOC patients did not contact Cancer Genetic Clinic and, consequently, did not undergo germline testing within our study’s observation period. Four of these patients (11.8%) carried a possible germline PV/LPV detected in the *BRCA1*, *RAD51C*, *BRIP1*, or *PTEN* gene in the tumor sample. If germline genotyping only was performed, 58.8% (30/51) PV/LPV in HBOC genes would be detected. In this case, 4 PV/LPV in HBOC genes could be missed due to patients’ unresponsiveness to genetic counseling/testing together with 17 somatic PV/LPV in HBOC genes. If tumor genotyping only was performed, 96.1% (49/51) PV/LPV in HBOC genes would be detected while 2 germline HBOC PV/LPV would be missed (1 × *BRCA1*, 1 × *ATM*) (Scheme 1 and Scheme 2). Similar conclusions were reached when only actionable PV/LPV for PARP inhibitor therapy were considered. With germline genotyping only, 68.4% (26/38) PV/LPV in BRCA genes would be detected, while with tumor genotyping only, 97.4% (37/38) PV/LPV in BRCA genes would be detected (Tables S1 and S2). Since we have detected potentially actionable PV/LPV for PARP inhibitor therapy in *BRIP1* and *RAD51C* genes, the final percentage of actionable detected PV/LPV with germline genotyping was 66.6% (28/42), and with tumor genotyping 97.6% (41/42) (Scheme 1 and Scheme 2).

## 5. Conclusions

Considering the facts that tumor testing enables detection of somatic and most germline PV/LPV, and that not all patients attend genetic counseling and testing, testing the tumor first seems to be a reasonable approach for detection of PV/LPV in HBOC genes in non-mucinous EOC patients. According to our findings, there is a high probability that non-mucinous EOC patients without PV/LPV in these genes in tumors would be negative also after germline testing. However, one should not forget that false negative results for germline PV/LPV are possible when performing genotyping of tumor tissue from FFPE samples, particularly in the case of large intragenic deletions.

For the purpose of accurate detection of germline and clinically actionable somatic PV/LPV in non-mucinous EOC patients, our proposed workflow would be as follows: NGS genotyping of tumor samples first, followed in tumor positive cases by NGS or Sanger sequencing for determination of origin of PV/LPV (germline or somatic) and genetic counseling. In negative cases, the decision whether the patient is referred to genetic counseling and testing should be based on additional criteria such as personal and family history, histological type of tumor, and its molecular characteristics. Such an approach would enable a timely identification of nearly all germline and somatic PV/LPV, whilst reducing the workload and costs associated with simultaneous testing of blood and tumor samples.

## Figures and Tables

**Table 1:** Pathogenic/likely pathogenic variants (PV/LPV) in HBOC genes among all 170 consecutive advanced non-mucinous EOC patients.

Group/Subgroup No. of Patients	PV/LPV (No.)	Patients with PV/LPV (No., %)	Germline PV/LPV (No.)	Patients with Germline PV/LPV (No., %)	Somatic PV/LPV (No.)	Patients with Somatic PV/LPV (No., %)	PV/LPV with Unknown Origin * (No.)	Patients with PV/LPV with Unknown Origin * (No., %)
Entire study group 170	51	46 (27.1%)	30 ***	29 (17.1%)	17	15 (8.8%)	4	4 (2.3%)
1. patients with unsuccessful tumor genotyping ** 6	0	0	0	0	NA	NA	NA	NA
2. patient with successful tumor and germline genotyping 132	47	42 (31.8%)	30 ***	29 (21.9%)	17	15 (11.3%)	0	0
3. non-responders 32	4	4	NA	NA	NA	NA	4	4 (12.5%)

%—percent of patients within the group/sub-group; PV/LPV—pathogenic/likely pathogenic variants; *—PV/LPV with unknown origin were found in patients that did not refer to genetic counseling and confirmatory germline testing; **—among 6 patients with unsuccessful tumor genotyping, no PV/LPV was found by germline genotyping (Scheme 1); ***—only 28 germline PV/LPV were detected by tumor genotyping; NA—not applicable.

**Table 2:** Pathogenic/likely pathogenic variants (PV/LPV) in BRCA genes among all 170 consecutive advanced non-mucinous EOC patients.

Group/Subgroup No. of Patients	PV/LPV (No.)	Patients with PV/LPV (No., %)	Germline PV/LPV (No.)	Patients with Germline PV/LPV (No., %)	Somatic PV/LPV (No.)	Patients with Somatic PV/LPV (No.,%)	PV/LPV with Unknown Origin * (No.)	Patients with PV/LPV with Unknown Origin * (No., %)
Entire study group 170	38	37 (21.8%)	26	26 (15.3%) ***	11	11 (6.5%) ***	1	1 (0.6%)
1. patients with unsuccessful tumor genotyping ** 6	0	0	0	0	NA	NA	NA	NA
2. patient with successful tumor and germline genotyping 132	37	36 (27.2)	26	26 (19.6%) ***	11	11 (8.3%) ***	0	0
3. non-responders 32	1	1 (3.1%)	NA	NA	NA	NA	1	1 (3.1%)

%—percent of patients within the group/sub-group; PV/LPV—pathogenic/likely pathogenic variants; *—PV/LPV with unknown origin were found in patients, that did not refer to genetic counseling and confirmatory germline testing; **—among 6 patients with unsuccessful tumor genotyping, no PV/LPV was found by germline genotyping (Scheme 1); ***—one patient is a carrier of somatic and germline PV/LPV in *BRCA2*, and one patient is carrier of somatic PV/LPV in *BRCA2* and germline PV/LPV in *CHEK2.* There are 10 patients carrying only somatic BRCA PV/LPV (without germline BRCA PV/LPV); NA—not applicable.

**Table 3:** Pathogenic/likely pathogenic variants (PV/LPV) in nonBRCA HBOC genes among all 170 consecutive advanced non-mucinous EOC patients.

Group/Subgroup No. of Patients	PV/LPV (No.)	Patients with PV/LPV (No., %)	Germline PV/LPV (No.)	Patients with Germline PV/LPV (No., %)	Somatic PV/LPV (No.)	Patients with Somatic PV/LPV (No., %)	PV/LPV with Unknown Origin * (No.)	Patients with PV/LPV with Unknown Origin * (No., %)
Entire study group. 170	13	11 (6.4%)	4 ***	3 (1.7%)	6 ****	5 (2.9%)	3 *****	3 (1.7%)
1. patients with unsuccessful tumor genotyping ** 6	0	0	0	0	NA	NA	NA	NA
2. patient with successful tumor and germline genotyping 132	10	8 (6.0%)	4 ***	3 (2.2%)	6 ****	5 (3.8%)	0	0
3. non-responders 32	3	3 (9.3%)	NA	NA	NA	NA	3 *****	3 (9.3%)

%—percent of patients within the group/sub-group: PV/LPV—pathogenic/likely pathogenic variants; *—PV/LPV with unknown origin were found in patients that did not refer to genetic counseling and confirmatory germline testing; **—among 6 patients with unsuccessful tumor genotyping, no PV/LPV was found by germline genotyping (Scheme 1); ***—germline PV/LPV in *RAD51C*, *ATM*, *BRIP1*, and *CHEK2*; ****—somatic PV/LPV in *NF1* (5 variants), and in *BARD1* (1 variant), one somatic PV/LPV in the *NF1* gene was found in patient harboring at the same time germline PV/LPV in the *BRCA1* gene and two somatic PV/LPV in *NF1* were found in one patient (Tables S1 and S2); *****—one in *RAD51C*, one in *BRIP1* and one in *PTEN* found in non-responders to genetic counseling/testing (Scheme 1); NA—not applicable.

**Table 4:** Comparison of tumor and germline genotyping results, for germline PV/LPV in BRCA genes, of matched tumor blood samples from the cohort of 132 advanced non-mucinous EOC patients, who had successfully genotyped tumors and blood samples.

Matched Tumor Samples	Matched Blood Samples		
	No. of patients–carriers of germline PV/LPV in BRCA genes	No. of patients with germline wild type BRCA genes	No. of all patients with genotyped matched blood sample
No. patients with PV/LPV in BRCA genes detected in matched tumor sample	25	10	35
No. patients with wild type BRCA genes in matched tumor sample	1 *	96	97
No. of all patients with genotyped matched tumor samples	26	106	132

PV/LPV—pathogenic/likely pathogenic variants; * patient with undetected large deletion in the *BRCA1* gene (exon 1–2 deletion).

**Table 5:** Comparison of tumor and germline genotyping results, for germline PV/LPV in HBOC genes, of matched tumor–blood samples from the cohort of 132 advanced non-mucinous EOC patients, who had successfully genotyped tumors and blood samples.

Matched Tumor Samples	Matched Blood Samples		
	No. of patients–carriers of germline PV/LPV in HBOC genes	No. of patients with germline wild type HBOC genes	No. of all patients with genotyped matched blood samples
No. patients with PV/LPV in HBOC genes detected in matched tumor samples	27	13 **	40
No. patients with wild type HBOC genes in matched tumor sample	2 *	90	92
No. of all patients with genotyped matched tumor samples	29	103	132

* Two non-mucinous EOC patients with germline pathogenic/likely pathogenic variants (PV/LPV) were not detected by tumor genotyping—one patient with *BRCA1* large deletion and another patient with SNV PV/LPV in *ATM* (indeed this patient was carrier of two germline mutations, another one in *RAD51C*, which was detected also by tumor genotyping); ** in one patient two somatic PV/LPV in NF1 were detected. Somatic PV/LPV were detected also in three patients with germline PV/LPV in HBOC genes.

## Data Availability

Data supporting reported results are available upon request.

## Supplementary Materials

The following supporting information can be downloaded at: https://www.mdpi.com/article/10.3390/cancers14061434/s1, Table S1: Detected pathogenic/likely pathogenic variants (PV/LPV) in HBOC genes in BRCA positive non-mucinous EOC patients (from the cohort all 170 patients included in the study). Results of tumor and blood (germline) genotyping, Table S2: Detected pathogenic/likely pathogenic variants (PV/LPV) in HBOC genes in BRCA negative non-mucinous EOC patients (from the cohort all 170 patients included in the study). Results of tumor and blood (germline) genotyping, Table S3: Number of germline pathogenic/likely pathogenic variants (PV/LPV) in BRCA and other HBOC genes detected in tumors of 132 non-mucinous EOC patients (from cohort with matched blood-tumor samples) according to variant type (SNV, small indels, large intragenic deletions), Table S4: Number of procedures performed in different testing approaches; germline testing first (scenario 1) and tumor genotyping first (scenario 2) compared to simultaneous tumor and germline genotyping (scenario 3), Table S5: Summary of two testing approaches: germline testing first (scenario 1) and tumor genotyping first (scenario 2).
