# Supplementary material for: Real-World Data on Detection of Germline and Somatic Pathogenic/Likely Pathogenic Variants in BRCA1/2 and Other Susceptibility Genes in Ovarian Cancer Patients Using Next Generation Sequencing
Source: Cancers (Basel). 2022 Mar 10;14(6):1434. doi: 10.3390/cancers14061434 (PMC8946582; doi:10.3390/cancers14061434)
Supplement: Supplementary file 1 [file cancers-14-01434-s001.zip › cancers-1614158-supplementary.pdf]

cancers

Supplementary Material: Real-World Data on Detection of Germline and Somatic Pathogenic/Likely Pathogenic Variants in *BRCA1*/2 and Other Susceptibility Genes in Ovarian Cancer Patients Using Next Generation Sequencing

Vida Stegel, Ana Blatnik, Erik Škof, Vita Šetrajčič Dragoš, Mateja Krajc, Brigita Gregorič, Petra Škerl, Ksenija Strojnik, Gašper Klančar, Marta Banjac, Janez Žgajnar, Maja Ravnik and Srdjan Novaković

Table S1. Detected pathogenic/likely pathogenic variants (PV/LPV) in HBOC genes in BRCA positive non-mucinous EOC patients (from the cohort of all 170 patients included in the study). Results of tumor and blood (germline) genotyping.

Sample No.	Percent of TC (%)	VAF in Tumor (%)	Tumor Genotyping Result	Blood Genotyping Result	OC or BC Family History
1	100	72	BRCA1:c.3436_3439delTGTT p.(Cys1146Leufs*8)	BRCA1:c.3436_3439delTGTT p.(Cys1146Lysfs*8)	yes
2	95	91	BRCA1:c.1687C>T p.(Gnl563*)	BRCA1:c.1687C>T p.(Gln563*)	yes
3	70	61	BRCA1:c.1687C>T p.(Gln563*)	BRCA1:c.1687C>T p.(Gln563*)	no
4	100	28	BRCA1:c.1687C>T p.(Gln563*)	BRCA1:c.1687C>T p.(Gln563*)	no
		43	BRCA1:c.1687_1689delinsTAC p.(Gln563Tyr) (RM)	wt for this variant	
5	90	46	BRCA2:c.6398C>G p.(Ser2133*)	BRCA1/2:wt	yes
6	90	57	BRCA1:c.1687C>T p.(Gln563*)	BRCA1:c.1687C>T p.(Gln563*)	yes
7	100	40	BRCA1: deletion of part of exon 22 and 23 c.5461_(5592_?)del p.?	BRCA1/2:wt	yes
8	80	62	BRCA1:c.844_850dupTCATTAC p.(Gln284Leufs*5)	BRCA1:c.844_850dupTCATTAC p.(Gln284Leufs*5)	yes
9	25	73	BRCA2:c.1813dup p.(Ile605Asnfs*11)	BRCA2:c.1813dupA p.(Ile605Asnfs*11)	yes
10	95	69	BRCA2:c.5291C>G p.(Ser1764*)	BRCA2:c.5291C>G p.(Ser1764*)	no
11	85	86	BRCA1:c.2377A>T p.(Lys793*)	BRCA1:c.2377A>T p.(Lys793*)	no
12	70	74	BRCA1:c.1687C>T p.(Gln563*)	BRCA1:c.1687C>T p.(Gln563*)	no
13	55	46	BRCA2:c.7806-2A>G p.?	BRCA2:c.7806-2A>G p.?	yes
		9	BRCA2:c.9502_9503insTA p.(Asn3168Ilefs*50)	wt for this variant	
14	40	55	BRCA1:c.1687C>T p.(Gln563*)	BRCA1:c.1687C>T p.(Gln563*)	yes
15	90	86	BRCA1:c.181T>G p.(Cys61Gly)	BRCA1:c.181T>G p.(Cys61Gly)	no
16	60	27	BRCA1:c.4234_4246dupGCTGAACTAGAAG p.(Ala1416Glyfs*2)	BRCA1/2:wt	yes
17	30	5	BRCA2:c.1762delA p.(Ser588Alafs*)	BRCA1/2:wt	no
18	80	45	BRCA2:c.6352_6353delGT p.(Val2118Lysfs*10)	BRCA1/2:wt	no
19	90	79	BRCA2:c.5291C>G p.(Ser1764*)	BRCA2:c.5291C>G p.(Ser1764*)	yes
20	95	60	BRCA2: deletion of exons 14-21 c.(7007+1_7008-1)_(*1_?)del p.?	BRCA1/2:wt	no
		15	CHEK2:c.1100delC p.(Thr367Metfs*15)	CHEK2:c.1100delC p.(Thr367Metfs*15)	no
21	60	60	BRCA1: deletion of exons 4-9 c.(134+1_135-1)_(670+1_671-1)del p.?	BRCA1:deletion of exons 4-9 c.(134+1_135-1)_(670+1_671-1)del p.?	yes
22	90	67	BRCA1:c.5266dupC p.(Gln1756Profs*74)	BRCA1:c.5266dupC p.(Gln1756Profs*74)	no
23	100	80	BRCA1:deletion of exons 4-9 c.(134+1_135-1)_(670+1_671-1)del p.?	BRCA1: deletion of exons 4-9 c.(134+1_135-1)_(670+1_671-1)del p.?	yes

24	80	74	BRCA1:c.181T>G p.(Cys61Gly)	BRCA1:c.181T>G p.(Cys61Gly)	no
25	80	68	BRCA1:c.80+5G>A p.?	BRCA1:c.80+5G>A p.?	no
26	95	76	BRCA2:c.4284dupT p.(Gln1429Serfs*9)	BRCA2:c.4284dupT p.(Gln1429Serfs*9)	no
27	100	73	BRCA2:c.1358delC p.(Pro453Glnfs*7)	BRCA1/2:wt	no
28	90	75	BRCA1:c.5266dupC p.(Gln1756Profs*74)	BRCA1:c.5266dupC p.(Gln1756Profs*74)	no
29	90	78	BRCA1:c.2732delG p.(Gly911Glufs*89)	BRCA1/2:wt	no
30	90	84	BRCA1:c.3436_3439delTGTT p.(Cys1146Leufs*8)	BRCA1:c.3436_3439delTGTT p.(Cys1146Leufs*8)	no
31	100	46	BRCA1:c.5135G>A p.(Trp1712*)	BRCA1/2:wt	yes
32	95	61	BRCA1:c.3705_3708delCAAT p.(Asn1235Lysfs*28)	BRCA1/2:wt	no
33	95	60	BRCA1:deletion of exons 4-9 c.(134+1_135-1)_(670+1_671-1)del p.?	BRCA1:deletion of exons 4-9 c.(134+1_135-1)_(670+1_671-1)del p.?	yes
		25	NF1:c.587-2A>G p.?	NF1:wt	
34	80	84	BRCA2:c.2372delC p.(Ser791Tyrfs*19)	BRCA2:c.2372delC p.(Ser791Tyrfs*19)	no
35	90	65	BRCA1:c.116G>A p.(Cys39Tyr)	BRCA1:c.116G>A p.(Cys39Tyr)	no
36	30	ND	BRCA1/2:wt	BRCA1:deletion of exons 1-2 c.(?_-1)_(80+1_81-1)del p.?	yes
37	50	57	BRCA1:c.1687C>T p.(Gln563*)	not tested	unknown

Non-mucinous EOC patients were considered BRCA positive, if PV/LPV in BRCA genes was detected in their blood or tumor sample. PV/LPV—pathogenic/likely pathogenic variants; VAF—variant allele frequency; wt—wild type; wt for this variant—wild type for this variant (description used, when another PV/LPV was detected in the same gene); ND—not determined; OC—ovarian cancer; BC—breast cancer; unknown—germline status was not assessed due to unresponsiveness of patient to referral to genetic counseling and testing; RM—reverse mutation, which was not counted as PV/LPV. The remaining HBOC genes, not mentioned in the Table, were wild type with no PV/LPV found. In the columns of genotyping, where PV/LPV in BRCA genes are described, the other undescribed BRCA gene was wild type.

Table S2. Detected pathogenic/likely pathogenic variants (PV/LPV) in HBOC genes in BRCA negative non-mucinous EOC patients (from the cohort of all 170 patients included in the study). Results of tumor and blood (germline) genotyping.

Sample No.	Percent of TC (%)	VAF in Tumor (%)	Tumor Genotyping Result	Blood Genotyping Result	OC or BC Family History
38	90	59	NF1:c.732dupA p.(Cys245Metfs*7)	NF1:wt	yes
39	100	78	RAD51C:c.572-1G>C p.?	RAD51C:c.572-1G>C p.?	no
		ND (<5)	ATM:wt	ATM:c.5932G>T p.(Glu1978*)	
40	80	14	NF1:c.5895delA p.(Lys1965Asnfs*26)	NF1:wt	no
		11	NF1:c.6064_6070dupGTGAAAT p.(Leu2024Cysfs*17)	NF1:wt	
41	80	24	BARD1:c.1935_1954del p.(Cys645*)	BARD1:wt	no
42	unknown	52	RAD51C:c.705+1G>A p.?	unknown	unknown
43	90	64	NF1:c.7907+1G>T p.?	NF1:wt	no
44	95	17	PTEN:c.507delC p.(Ser170Valfs*13)	unknown	unknown
45	90	78	BRIP1:c.924dupA p.(Ser309Ilefs*10)	BRIP1:c.924dupA p.(Ser309Ilefs*10)	no
46	<1	46	BRIP1:c.1066C>T p.(Arg356*)	unknown	unknown

Non-mucinous EOC patients were considered BRCA negative, if no PV/LPV in BRCA genes was detected in their blood nor tumor sample. PV/LPV— pathogenic/likely pathogenic variants; VAF—variant allele frequency; wt—wild type; OC—ovarian cancer; BC—breast cancer; ND—not determined; unknown—germline status was not assessed due to unresponsiveness of patient to referral to genetic counseling and testing. The remaining HBOC genes, not mentioned in the Table, were wild type with no PV/LPV found.

Table S3. Number of germline pathogenic/likely pathogenic variants (PV/LPV) in BRCA and other HBOC genes detected in tumors of 132 non-mucinous EOC patients (from cohort with matched blood-tumor samples) according to variant type (SNV, small indels, large intragenic deletions).

Type of Germline PV/LPV Variant	No. of Germline PV/LPV Detected in Blood	No. of Germline PV/LPV Detected in FFPE Tumor Tissue	No. of Germline PV/LPV Overlooked in Corresponding FFPE Tumor Tissue
PV/LPV in BRCA genes			
SNV or 1bp deletion or insertion in BRCA genes	19	19	0
2-7bp deletion or insertion in BRCA genes	3	3	0
large intragenic deletions in BRCA genes	4	3	1*
Sum of all PV/LPV BRCA genes	26	25	1
PV/LPV in nonBRCA HBOC genes			
SNV or 1bp deletion or insertion in non BRCA HBOC genes	4	3	1
2-7bp deletion or insertion non BRCA HBOC genes	0	0	0
large intragenic deletions in non BRCA HBOC genes	0	0	0
Sum of all PV/LPV in non BRCA HBOC genes	4	3	1
Sum of all PV/LPV HBOC variants	30	28	2

Table S4. Number of procedures performed in different testing approaches; germline testing first (scenario 1) and tumor genotyping first (scenario 2) compared to simultaneous tumor and germline genotyping (scenario 3).

Procedures	Scenario 1 (S1) *	Scenario 2 (S2) **	Scenario 3 (S3) ***
No. and % of pre-test genetic counseling	138 (100%)	77 (56%)	138 (100%)
No. and % post-test genetic counseling	138 (100%)	77 (56%)	138 (100%)
No. and % NGS germline genotyping	138 (100%)	33 (24%)	138 (100%)
No. and % germline confirmatory testing (Sanger or MLPA) ****	60 (100%)	50 (83%)	60 (100%)
No. and % NGS tumor genotyping	109 (64%)	170 (100%)	170 (100%)
Time for >95% of tested patients to reach full testing result	4–6 weeks	2–3 weeks	2–3 weeks
% of detected patient with PV/LPV in BRCA genes either tumor or germline genotyping result after 3 weeks	68%	97%	100%

PV/LPV—pathogenic/likely pathogenic variants;* in germline testing first (scenario 1) NGS tumor genotyping is performed only in germline PV/LPV negative cases; ** in tumor genotyping first (scenario 2) NGS germline genotyping is performed only in tumor PV/LPV negative cases with a family history of HBOC cancers; *** in simultaneous tumor and germline genotyping (scenario 3) in all cases; **** all PV/LPV detected in patients are confirmed with MLPA or Sanger sequencing after additional blood sampling. NGS tumor genotyping is performed, and NGS germline is performed in all cases, where patients responded to referral to genetic counseling.

Table S5. Summary of two testing approaches: germline testing first (scenario 1) and tumor genotyping first (scenario 2).

Procedures	Scenario 1 (S1) *	Scenario 2 (S2) **	Difference between S1 and S2 No (%)
No. of pre-test genetic counseling	138	77	↓61 (−44%)
No. of post-test genetic counseling	138	77	↓61 (−44%)
No. of NGS germline genotyping	138	33	↓105 (−76%)
No. of germline confirmatory testing (Sanger or MLPA) ***	60	50	↓13 (−16%)

No. of NGS tumor genotyping	109	170	↑61 (+56%)
Time for >95% of tested patients to reach full testing result	4-6 weeks	2-3 weeks	↓2–3 weeks (−50%)
% of detected patient with PV/LPV in BRCA genes either tumor or germline genotyping result after 3 weeks	68%	97%	↑+29%

↓—decrease; ↑—increase; PV/LPV—pathogenic/likely pathogenic variants; * in germline testing first (scenario 1) NGS tumor genotyping is performed only in germline PV/LPV negative cases; ** in tumor genotyping first (scenario 2); *** all PV/LPV detected in patients are confirmed with MLPA or Sanger sequencing after additional blood sampling. NGS germline genotyping is performed only in tumor PV/LPV negative cases having family history of HBOC cancers.
